# Airport smoking zones: a prospective public health hazard

**DOI:** 10.1097/JS9.0000000000000159

**Published:** 2023-03-24

**Authors:** Ravinder Singh, Manjinder Singh, Varinder Singh, Pratima Kumari, Hitesh Chopra, Priyanka, Om Prakash Choudhary, Talha B. Emran

**Affiliations:** a Chitkara College of Pharmacy, Chitkara University, Punjab. India; b Department of Veterinary Microbiology, College of Veterinary Science, Guru Angad Dev Veterinary and Animal Sciences University (GADVASU), Rampura Phul, Bathinda, Punjab; c Department of Veterinary Anatomy and Histology, College of Veterinary Sciences and Animal Husbandry, Central Agricultural University (I), Selesih, Aizawl, Mizoram, India; d Department of Pharmacy, BGC Trust University Bangladesh; e Department of Pharmacy, Faculty of Allied Health Sciences, Daffodil International University, Dhaka, Bangladesh

Highlights

- Infections might happen in crowded and inadequately ventilated spaces.
- Tobacco smoking can severely damage the respiratory organs.
- WHO Global report, 9% of deaths occur due to tobacco smoking.
- Chronic exposure to smoke and the release of these inflammatory mediators.

*Dear Editor*,

As per the WHO, the infections might happen in crowded and inadequately ventilated spaces like airports through aerosolized or very small droplets. Doctors have expressed apprehensions that small, poorly ventilated designated smoking areas, as at airports, can accelerate the spread of infectious diseases, particularly respiratory infections^1^. In fact, the particulate matter (PM) of air inside these designated smoking areas at airports is 15 times higher than that the recommended by WHO (10 µg/m^3^) which further poses a higher risk of spread of infections among people smoking tobacco at these confined place^2^.

Tobacco smoking can severely damage the respiratory organs and is one of the major contributing factors to respiratory infection^3^. Tobacco smoke contains more than 7000 compounds of which nicotine, a major component that is well known to develop tobacco addiction^4^, and other 200 (such as benzpyrene, arsenic, and cadmium) compounds are identified as noxious and oncogenic as they may irritate the respiratory tract and reduce the erythrocyte’s oxygen transport capacity^5^.

Smoking rooms in the four largest international airports with the most operating designated smoking rooms (DSRs) were observed using PM2.5 monitoring equipment followed by an approved research protocol for evaluating fine particle pollution from tobacco smoke. The overall mean PM2.5 levels in the DSR were 532.5 mg/m^3^ which is extremely high and dangerous. The high levels of secondhand smoke (SHS) in and adjacent to DSR show that these rooms are not providing safe air quality for employees and travelers^6^. A study in 2010 found that PM2.5 which is a common measure of SHS, leaked from enclosed airport smoking venues and entered the general air circulation which exposed the workers and the public to SHS^7^. The average PM2.5 concentrations inside the smoking rooms were notably higher than the National Ambient Air Quality Standard for 24 hours (35 μg/m^3^)^8^.

Globally, smoking remains a major concern for public health. As per the WHO Global report, 9% of deaths occur due to tobacco smoking and about 50% of the smokers die from smoking-associated disorders. It is a well-known fact that smoking increases the risk and recurrence rate of respiratory infections including pneumococcal, tuberculosis, and other acute respiratory tract infections. Often, these are transmitted by a variety of pathogens particularly, bacteria (*Mycobacterium tuberculosis*), fungi and viruses (coronavirus, rhinovirus parainfluenza, etc.). The risk of community-acquired pneumonia among smokers is 1.5 times of the nonsmokers and thus smoking is identified as a concerning risk factor leading to invasive pneumococcal diseases. Smokers are also more prone to acute respiratory tract infections, with a longer cough duration (8.9 vs. 6.8 days) and an increased risk of lower respiratory illnesses (57 vs. 45%). The increased risk of influenza-related hospitalization (2.49 times than nonsmokers) is observed in smokers. Even, the treatment of influenza-affected smokers poses a big challenge as the efficacy of the influenza vaccine has been reported to be decreased dramatically in smokers which were recently been observed during the COVID-19 pandemic^9^.

Due to the complex pathological events, the mechanisms by which tobacco smoking-induced these infections are not fully understood. Fibrosis, inflammation, reduced mucociliary clearance, and deterioration of goblet cells, ciliated cells, submucosal secretary glands, and respiratory epithelium are the few anatomical changes that are indicated to be induced by tobacco smoke and its components. This makes the smokers susceptible to upper and lower respiratory tract infections. Also, smoking modulates both cell-mediated (30% higher peripheral white blood count) and humoral-mediated (up to 20% lower serum immunoglobulin levels) immune responses which may further intensify airway hyper-reactivity and inflammation. It has been found that cigarette smoke augments the generation of a variety of pro-inflammatory cytokines including tumor necrosis factor-α, interleukins (IL-1, 6, and 8), white blood cell, and C-reactive protein^10^. Chronic exposure to smoke and the release of these inflammatory mediators by neutrophils and macrophages would result into chronic obstructive pulmonary disease and its complications.

Besides lungs, deterioration of oral health is also a major concern in smokers. Epidemiological studies have shown a positive correlation between tobacco smoking and inflammatory diseases of oral tissues such as salivary glands, mucosa, and periodontium. Surprisingly, smokers are up to five times more prone to periodontal disorders. Reactive oxygen species generated by smoke triggers the release of inflammatory mediators like tumor necrosis factor-α, ILs, and matrix metalloproteinases which damage the periodontal tissues by degrading the extracellular matrix proteins^11^.

Although the sale of the tobacco products is one of the prominent sources of revenue for the governments, tobacco smoking is associated with 50% of mortality among smokers and thus places a heavy toll on healthcare systems. Progress has been made in improving the air quality of smoking designated places at airports by strict implementation of concerned policies^12^. To contain the spread of infections, the government are emphasizing on absolute smoke-free airports in their respective nations. In fact, countries like Australia and New Zealand have already implemented such regulations to shield the spread of smoke-induced infections (Fig. 1).

**Figure 1:**
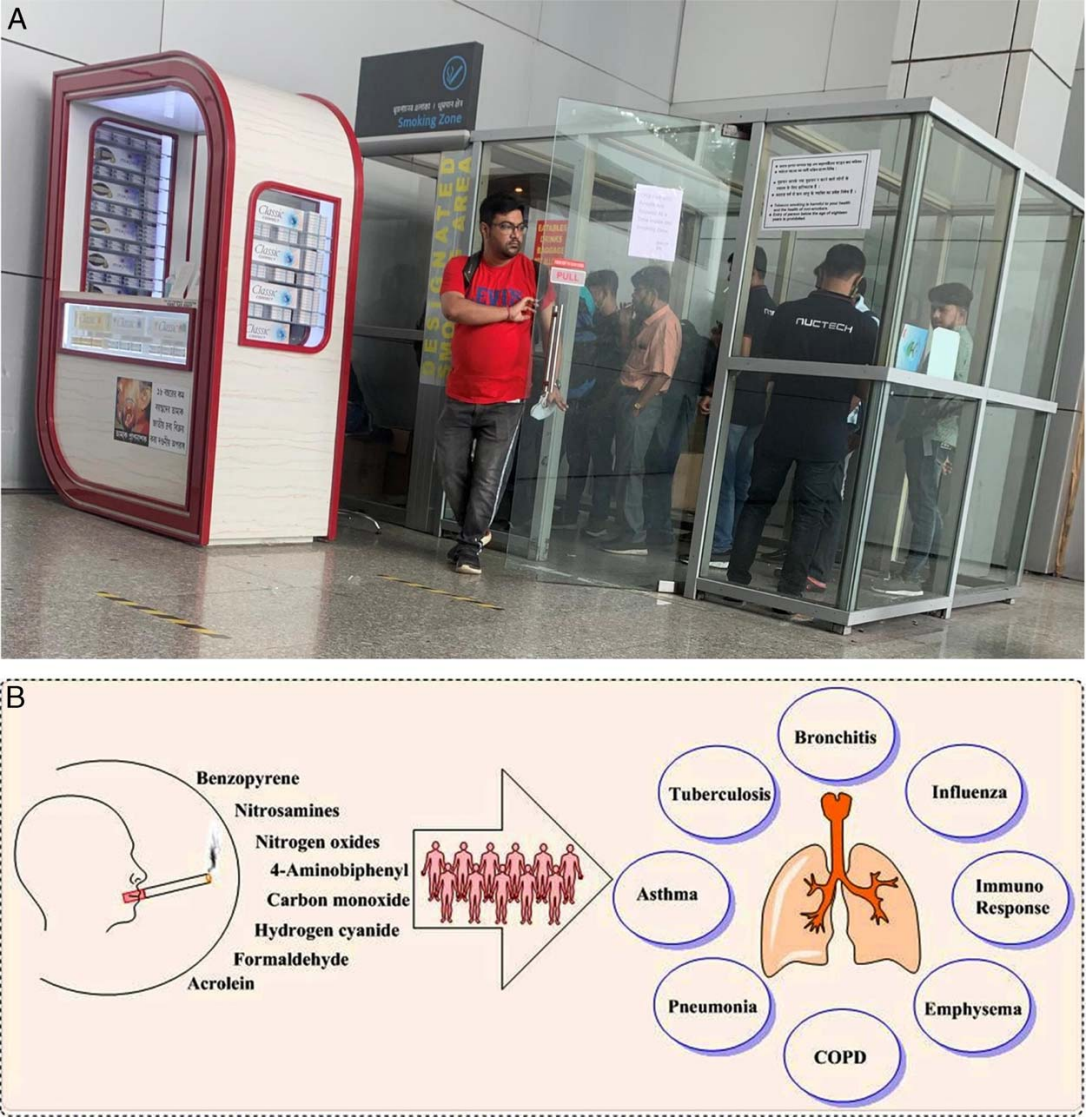
(A) Gathering of people at the smoking zone at the Netaji Subhash Chandra Bose International Airport, Kolkata, India. (B) Major components in tobacco used in cigarettes and several plausible threats due to smoking. COPD, chronic obstructive pulmonary disease.

## Ethical approval

Not applicable.

## Sources of funding

None.

## Authors’ contribution

R.S.: conceptualization, data curation, writing – original draft preparation, writing – reviewing and editing. M.S., P.K., H.C., Priyanka, and O.P.C.: data curation, writing – original draft preparation, writing – reviewing and editing. V.S.: data curation, writing – original draft preparation, writing – reviewing and editing, visualization, supervision. T.B.E.: writing – reviewing and editing, visualization, supervision.

## Conflicts of interest disclosure

The authors declare that they have no financial conflict of interest with regard to the content of this report.

## Research registration unique identifying number (UIN)

Not applicable.

## Guarantor

Talha B. Emran.

## Provenance and peer review

Not commissioned, internally peer-reviewed.
